# Engineering yeast mitochondrial metabolism for 3-hydroxypropionate production

**DOI:** 10.1186/s13068-023-02309-z

**Published:** 2023-04-08

**Authors:** Yiming Zhang, Mo Su, Yu Chen, Zheng Wang, Jens Nielsen, Zihe Liu

**Affiliations:** 1 Beijing Advanced Innovation Center for Soft Matter Science and Engineering, College of Life Science and Technology, Beijing University of Chemical Technology, Beijing, 100029 China; 2 Department of Biology and Biological Engineering, Chalmers University of Technology, SE-412 96 Gothenburg, Sweden; 3 BioInnovation Institute, Ole Maaløes Vej 3, DK2200 Copenhagen, Denmark

**Keywords:** Yeast mitochondrion, Malonyl-CoA reductase, 3-Hydroxypropionate, Redox factor engineering, Acetyl-CoA carboxylase

## Abstract

**Background:**

With unique physiochemical environments in subcellular organelles, there has been growing interest in harnessing yeast organelles for bioproduct synthesis. Among these organelles, the yeast mitochondrion has been found to be an attractive compartment for production of terpenoids and branched-chain alcohols, which could be credited to the abundant supply of acetyl-CoA, ATP and cofactors. In this study we explored the mitochondrial potential for production of 3-hydroxypropionate (3-HP) and performed the cofactor engineering and flux control at the acetyl-CoA node to maximize 3-HP synthesis.

**Results:**

Metabolic modeling suggested that the mitochondrion serves as a more suitable compartment for 3-HP synthesis via the malonyl-CoA pathway than the cytosol, due to the opportunity to obtain a higher maximum yield and a lower oxygen consumption. With the malonyl-CoA reductase (MCR) targeted into the mitochondria, the 3-HP production increased to 0.27 g/L compared with 0.09 g/L with MCR expressed in the cytosol. With enhanced expression of dissected MCR enzymes, the titer reached to 4.42 g/L, comparable to the highest titer achieved in the cytosol so far. Then, the mitochondrial NADPH supply was optimized by overexpressing *POS5* and *IDP1*, which resulted in an increase in the 3-HP titer to 5.11 g/L. Furthermore, with induced expression of an *ACC1* mutant in the mitochondria, the final 3-HP production reached 6.16 g/L in shake flask fermentations. The constructed strain was then evaluated in fed-batch fermentations, and produced 71.09 g/L 3-HP with a productivity of 0.71 g/L/h and a yield on glucose of 0.23 g/g.

**Conclusions:**

In this study, the yeast mitochondrion is reported as an attractive compartment for 3-HP production. The final 3-HP titer of 71.09 g/L with a productivity of 0.71 g/L/h was achieved in fed-batch fermentations, representing the highest titer reported for *Saccharomyces cerevisiae* so far, that demonstrated the potential of recruiting the yeast mitochondria for further development of cell factories.

**Supplementary Information:**

The online version contains supplementary material available at 10.1186/s13068-023-02309-z.

## Background

The yeast *Saccharomyces cerevisiae* has been extensively engineered for synthesis of biofuels and other non-ethanol bioproducts [1, 2]. Most synthetic pathways are targeted to the cytosol due to its big volume, high coverage of metabolic enzymes and metabolites [3, 4]. Recent studies have revealed that the yeast mitochondrion could be attractive to produce terpenoids, fumarate, ornithine, and branched-chain alcohols [5–7]. With the mevalonate pathway and sequential synthetic genes targeted into the mitochondria, researchers have greatly improved the titers of isoprene, patchoulol, valencene, amorphadiene, linalool and santalene [8–12]. Moreover, mitochondrial compartmentalization of the 2-ketoacid elongation pathway has obtained 1.24 g/L isopentanol, the highest titer reported in yeast [13]. Therefore, there has been growing interest in exploring the potential of mitochondrial compartmentalization of synthetic pathways.

The improved production of subcellular compartmentation may be ascribed to physical separation, which concentrates overexpressed enzymes and substrates in a smaller volume to reduce the amount of the byproducts or the toxicity of some intermediate metabolites. Specially for mitochondrial compartmentation, there are more acetyl-CoA, ATP and NADH available compared with the cytosol. Furthermore, the mitochondrial matrix has a higher pH and redox potential than the cytosol, and this environment could be more suitable for bacterial enzymes [14]. Taking together, the mitochondrial compartment could be a promising alternative for production of 3-hydroxypropionate (3-HP), which could be synthesized from acetyl-CoA via the malonyl-CoA pathway catalyzed by acetyl-CoA carboxylase (ACC) and a bacterial enzyme malonyl-CoA reductase (MCR). 3-HP serves as a promising platform chemical to produce various C3-based chemicals, including acrylic acid and its derivatives, which are wildly used in the fields of plastics, pharmaceuticals and agriculture. Extensive studies of 3-HP bioproduction have been performed [15–17]*.*

Previous studies on the malonyl-CoA pathway have been performed in yeast cytosol and revealed several crucial factors for 3-HP synthesis, including efficient supply of acetyl-CoA and malonyl-CoA, balanced supply of redox cofactor NADPH, and improved activities of MCR enzymes [18–20]. The native cytosolic acetyl-CoA synthesis is highly ATP demanding, and engineering efforts on acetyl-CoA biosynthetic pathways have been performed and enhanced 3-HP production by up to fivefold [21, 22]. Cytosolic malonyl-CoA is mainly involved in fatty acid synthesis, which is regulated stringently at multiple levels. The acetyl-CoA carboxylase Acc1 functions as one of the important nodes in fatty acid metabolism and is stringently regulated transcriptionally and post-translationally [23, 24]. With malonyl-CoA biosensor established in yeast, it has been found that overexpression, dynamic control and mutagenesis of dephosphorylation sites of Acc1 can direct more malonyl-CoA to 3-HP synthesis rather than fatty acid synthesis [25–27]. Combined with these strategies for enhanced precursor supply, engineering redox metabolism for optimized NADPH supply further improves 3-HP production [20–22]. Moreover, it has been reported that MCR engineering with domain dissection and balanced expression of the two dissected parts can significantly improve 3-HP production from 0.107 to 3.72 g/L in *E. coli* [28, 29]. With the dissected enzymes expressed in yeast cytosol, a significant improvement of 3-HP production is also achieved from 0.6 to 2.4 g/L, suggesting MCR as another limiting factor for 3-HP [18].

Compared with cytosolic precursor supply, the mitochondrial acetyl-CoA derived from pyruvate dehydrogenase (PDH) is used as fuel of the TCA cycle and is reported to be 10 times more concentrated than its cytosolic counterpart, which renders the mitochondria great potential for 3-HP synthesis. Mitochondrial acetyl-CoA can be converted to malonyl-CoA by Hfa1 [30, 31]. This mitochondrial isoform Hfa1 shares 72% sequence similarity with Acc1, and is essential for synthesis of mitochondrial fatty acids, especially lipoic acid cofactor [30]. Its initiation regulation is reported to be translated from non-canonical initial site with a mitochondrial targeting sequence at its N terminal [32]. So far, it remains unclear whether Hfa1 contains any regulation sites, and it would be interesting to investigate its effects on the synthesis of malonyl-CoA derived bioproduct.

While the cytosolic NADPH is mainly derived from the reactions catalyzed by glucose-6-phosphate dehydrogenase Zwf1, acetaldehyde dehydrogenase Ald6 and isocitrate dehydrogenase Idp2, the mitochondrial NADPH is relying on the reactions catalyzed by NAD^+^/NADH kinase Pos5 and NADP^+^ dependent enzymes, including acetaldehyde dehydrogenases Ald4 and Ald5, isocitrate dehydrogenase Idp1, and malic enzyme Mae1 [33, 34]. Pos5 is considered as the main source of mitochondrial NADPH and possesses higher NADH kinase activity than NAD kinase activity [35, 36], suggesting active NADPH synthesis from NADH with ATP consumed, both of which are abundant in the mitochondriona. Taking together, the yeast mitochondrion seems to be a suitable subcellular compartment for 3-HP production.

**Fig. 1:**
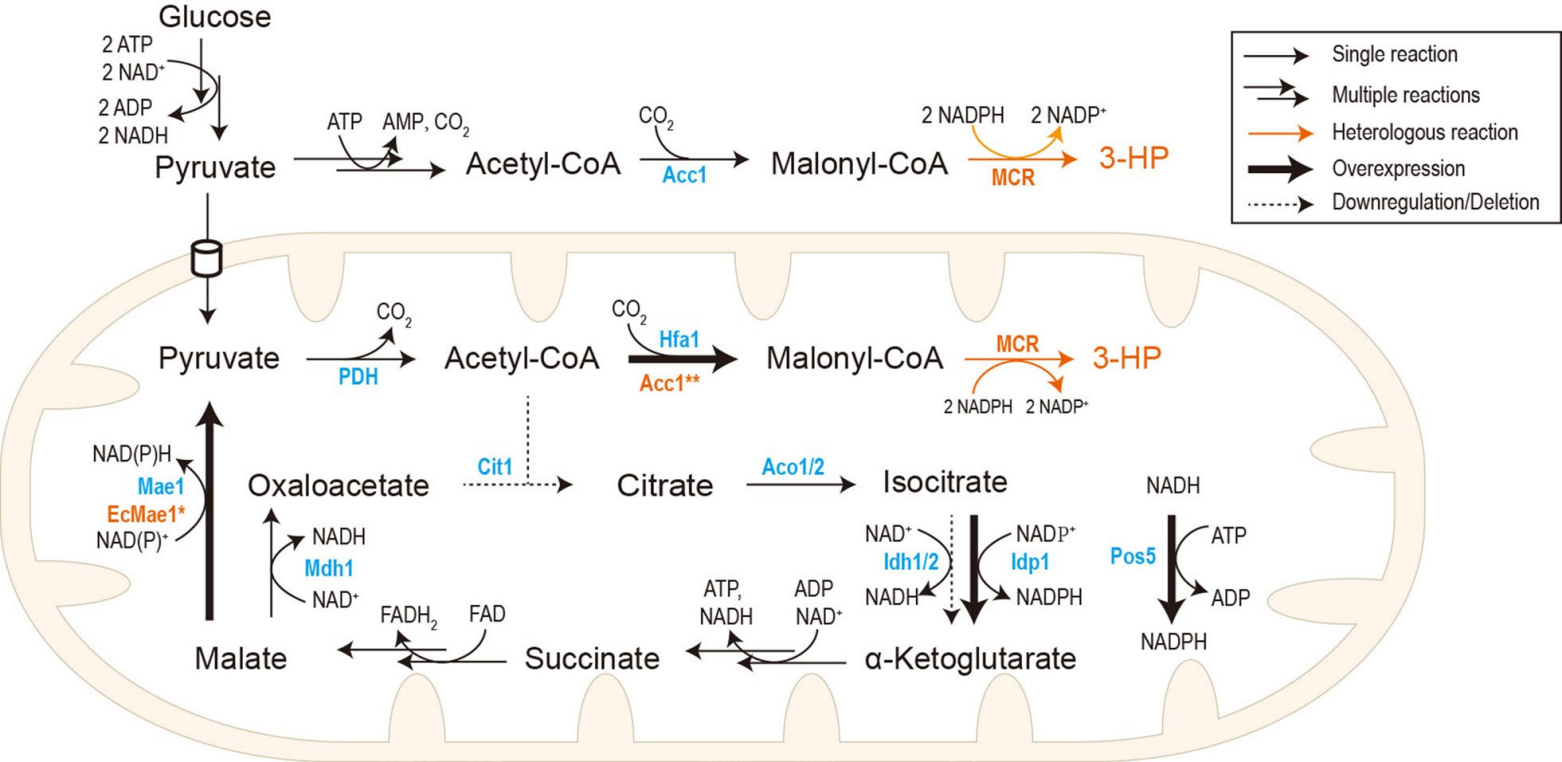
Schematic representation of rewiring mitochondrial metabolism for 3-HP production. The native enzymes were shown in blue, whereas heterologous enzymes in orange. *Acc1* acetyl-CoA carboxylase, *MCR* malonyl-CoA reductase, *PDH* pyruvate dehydrogenase complex, *Hfa1* mitochondrial acetyl-CoA carboxylase, Acc1** acetyl-CoA carboxylase mutant, *Cit1* mitochondrial citrate synthase, *Aco1/2* aconitase, *Idh1/2* mitochondrial NAD^+^-dependent isocitrate dehydrogenase, *Idp1* mitochondrial NADP-specific isocitrate dehydrogenase, *Mdh1* mitochondrial malate dehydrogenase, *Mae1* mitochondrial malic enzyme, EcMaeA* malic enzyme mutant from *E. coli*, *Pos5* mitochondrial NADH kinase

Here we explored yeast mitochondrion for its potential in 3-HP production via the malonyl-CoA pathway. Engineering efforts were performed to enhance both malonyl-CoA and NADPH supply (Fig. 1). The work presented here gains a better understanding for harnessing the yeast mitochondrion for synthesis of acetyl-CoA derived biochemicals, especially those with NADPH as a cofactor.

## Materials and methods

### Construction of plasmids and strains

The *E. coli* DH5α strain was used for plasmid construction. The yeast strain CEN.PK 113-5D was used as the background strain. Genetic manipulations, including gene integration, knockout and promoter replacement, were performed using the GTR-CRISPR system [37]. Plasmids used in the study were constructed with Golden Gate methods if not specified [38]. The strains and plasmids used and constructed were listed in Additional file 1.

The intact *MCR* from *Chloroflexus aurantiacus* was cloned in pMCR1, while its dissected parts of *MCR-N* and *MCR-C* were cloned in pMCR2 in a previous study for mitochondrial localization [39]. The plasmid pMCR-mC was constructed to introduce the mutations N940V, K1106W, and S1114R into *MCR-C* by assembling 3 fragments amplified with primers MP1–4 from pMCR2 [39] into the vector pUGG1. The plasmid pMCR3 was constructed by assembling the fragments of *MCR-N* amplified with primers MP5&6 and *MCR-mC* amplified with primers MP7&8 into pUGG1. The plasmid pPOS5 was constructed to overexpress *POS5* under the control of *TDH3* promoter. The *TDH3p* fragment amplified with primers MP9&10 and the fragment containing *POS5* and its terminator amplified with primers MP11&12 using yeast genomic DNA as the template were assembled into pUGG1. The plasmid pALD4 was constructed to overexpress *ALD4* under the control of *PGK1* promoter. The *PGK1p* fragment amplified with primers MP13&14 and the fragment containing *ALD4* and its terminator amplified with primers MP15&16 were assembled into pUGG1. The plasmid pIDP1 was constructed to overexpress *IDP1* under the control of *TEF1* promoter. The *TEF1p* fragment amplified with primers MP17&18 and the fragment containing *IDP1* its terminator amplified with primers MP19&20 were assembled into pUGG1. The plasmid pMaeA* was constructed to overexpress MaeA* from *E. coli* under the *TEF1* promoter. The fragments containing *TEF1p* and the mitochondrial tag *CoxIVm* were amplified from pSSB02 [40] with primers MP21–24. The mutated MaeA fragments were amplified with primers MP25–28 from the *E. coli* genomic DNA. The *ADH1t* fragment was amplified with primers MP29&30 from yeast genomic DNA. Then, these fragments were assembled into pUGG1.

Mitochondrial overexpression of *ACC1* was achieved using different promoters of varied strength and types, including *TEF1p, CYC1p, CUP1p* and *GAL1p*. The fragments containing *ACC1*^S659A, S1157A^ were amplified from pCfB376 [27] with primers MP31–35 and MP29. The fragments containing *TEF1*p and *CoxIV*m were amplified from pSSB02 with primers MP21–24. Then, the two fragments were assembled into pUGG1 to generate pACC1**_TEF1p. Then, *ACC1*^S659A, S1157A^ was mutated to the wild-type ACC1 by assembling two fragments amplified with primers MP36&37 and MP38&39, generating pACC1_TEF1p. Based on pACC1_TEF1p, *TEF1p* was replaced to *CYC1p* by assembling the fragment amplified with primers MP40–43 using the NEBuilder assembly to generate pACC1_CYC1p. Based on pACC1**_TEF1p, *TEF1p* and *2μ ori* were replaced to *CUP1pI* and *CEN4/6*, respectively, by assembling the fragments amplified with primers MP44–51 using the NEBuilder assembly to generate pACC1**_CUP1p. Based on pACC1_TEF1p and pACC1**_TEF1p, *TEF1p* was replaced to *GAL1p* using NEBuilder assembly to generate pACC1_GAL1p and pACC1**_GAL1p. Based on pACC1**_CUP1p, *CUP1p* was replaced to *GAL1p* using the NEBuilder assembly to generate pACC1**_GAL1p_CEN.

For genome integration, reported integrative sites were used, including XI-3, X-4 and Int14 [41–43]. Intact MCR was integrated at XI-3 using pCas9_XI-3.3 and donor DNA amplified from pMCR1 with primers MP54&55. Dissected MCR was integrated at XI-3 site using pCas9_XI-3.3 and donor DNA fragments amplified from pMCR2 or pMCR3 with primers MP55&56. MCR-N and MCR-mC were integrated at X-4 and Int14 with donor DNA fragments amplified with MP57&58, and MP60&61, respectively. Moreover, several native promoters were replaced with strong constitutive promoters to enhance their expression. *POS5p* was replaced to *TDH3p* using pCas9_POS5p and donor DNA amplified with MP63&64. *IDP1p* was replaced to *TEF1p* using pCas9_IDP1p and donor DNA amplified with MP66&67. *MAE1p* was replaced to *TDH3p* using pCas9_MAE1p and donor DNA amplified with MP69&70. *HFA1p* was replaced to *TEF1p* using pCas9_HFA1p and donor DNA amplified with MP72&73. Meanwhile, *IDH1* was deleted using pCas9_idh1 and donor DNA amplified with primers MP75&76. *IDH2* was deleted using pCas9_idh2 and donor DNA amplified with primers MP78&79. *CIT1* was deleted using pCas9_cit1 and donor DNA amplified with primers MP81&82. *GAL80* was deleted using pCas9_gal80 and donor DNA amplified with primers MP85&86.

### Media and cultivation condition

*E. coli* strains were cultivated in LB medium supplemented with 80 mg/L ampicillin when needed. Yeast strains were cultivated in YPD medium, or SC-URA medium to maintain *URA3*-marked plasmids, as previously described in [44]. Fermentation medium for 3-HP production in shake flasks was composed of 7.5 g/L (NH_4_)_2_SO_4_, 14.4 g/L KH_2_PO_4_, 0.5 g/L MgSO_4_·7H_2_O, 20 g/L glucose, trace metal solution and vitamin solution as described in [45] and 40 mg/L uracil was added when needed. For induction for the *CUP1* promoter, 300 μM CuSO_4_ was supplemented at 24 h during the fermentation. For all shake flask experiments, 20 mL cultures were cultivated in 100 mL shake flasks with the initial OD_600_ of 0.1 at 30 °C and 200 rpm.

For fed-batch cultivations in 5 L bioreactors, the medium was composed of 5 g/L (NH_4_)_2_SO_4_, 3 g/L KH_2_PO_4_, 0.5 g/L MgSO_4_·7H_2_O, 20 g/L glucose, as well as the trace metal solution and vitamin solution as described in [45]. The seed was pre-cultured using the same medium in the 500 mL shake flasks, and then inoculated into the bioreactors with 2 L medium with the initial OD_600_ of 0.4. During the fermentation, the dissolved oxygen (DO) concentration was controlled above 15% by adjusting the stirring speed within 200–700 rpm and aeration with 0.5–1 vvm. pH was controlled at 5.0 by feeding NH_3_·H_2_O, and the temperature was controlled at 30 °C. The feed medium was added when glucose was almost exhausted, composing 5.6 g/L K_2_SO_4_, 15 g/L KH_2_PO_4_, 2.8 g/L M_g_SO_4_·7H_2_O, 700 g/L glucose, trace metal solution (7 ×) and vitamins solution (7 ×), and the feed rates were continuously adjusted to remain low glucose residue and ethanol residue in the culture.

### Measurement of biomass and extracellular metabolites

The optical density was measured at 600 nm OD_600_ by GENESYS 30 Visible Spectrophotometer (Thermo Electron Scientific, Madison, USA).

Extracellular metabolites analysis was performed using the HPLC (Shimadzu LC-20AT, Japan) equipped with RID detector and PDA detector using Aminex HPX-87H column (Bio-Rad). The culture samples were centrifuged, and the supernatant was filtered by 0.22 μm membrane and stored in − 20 °C until analysis. The eluent of 0.5 mM H_2_SO_4_ was used at a flow rate of 0.5 mL/min with the column temperature of 65 °C for the measurement of glucose, ethanol and 3-HP. Glucose, ethanol and 3-HP were quantified using LabSolution Software (Shimadzu).

### Flux balance analysis

The maximum theoretical 3-HP yields with the malonyl-CoA pathway expressed in the cytosol and mitochondria were calculated using flux balance analysis (FBA) [46] with the yeast genome-scale metabolic model Yeast8 [47], which was correspondingly modified by adding the malonyl-CoA pathway in the cytosol and mitochondria. The glucose uptake rate was fixed at 1 mmol/g DCW/h and the 3-HP secretion rate was maximized, and the simulated flux distribution can be then used to determine the active reactions, i.e., those whose flux is non-zero. The simulations were performed in MATLAB using the Cobra toolbox v.3.0 [48], and the simulated flux distributions were then used to calculate the maximum theoretical yields.

## Results and discussion

### Mitochondrial localization of MCR enhanced 3-HP synthesis

Flux balance analysis was performed based on the genome scale metabolic model of *S. cerevisia*e Yeast8 [47], to calculate the maximum theoretical yields of 3-HP for cytosolic and mitochondrial synthesis. A theoretical 3-HP yield of 1.71 mol/mol glucose was obtained with the oxygen requirement of 0.88 mol/mol glucose for mitochondrial synthesis, compared with those of 1.62 mol 3-HP/mol glucose and 1.15 mol O_2_/mol glucose for cytosolic synthesis (Additional file 2). The higher theoretical yield and lower oxygen requirement could be credited to the rich acetyl-CoA and ATP source in the mitochondria, as cytosolic acetyl-CoA synthesis was highly ATP demanding, rendering mitochondrial synthesis great potentials for future engineering in both basic and applied research.

**Fig. 2:**
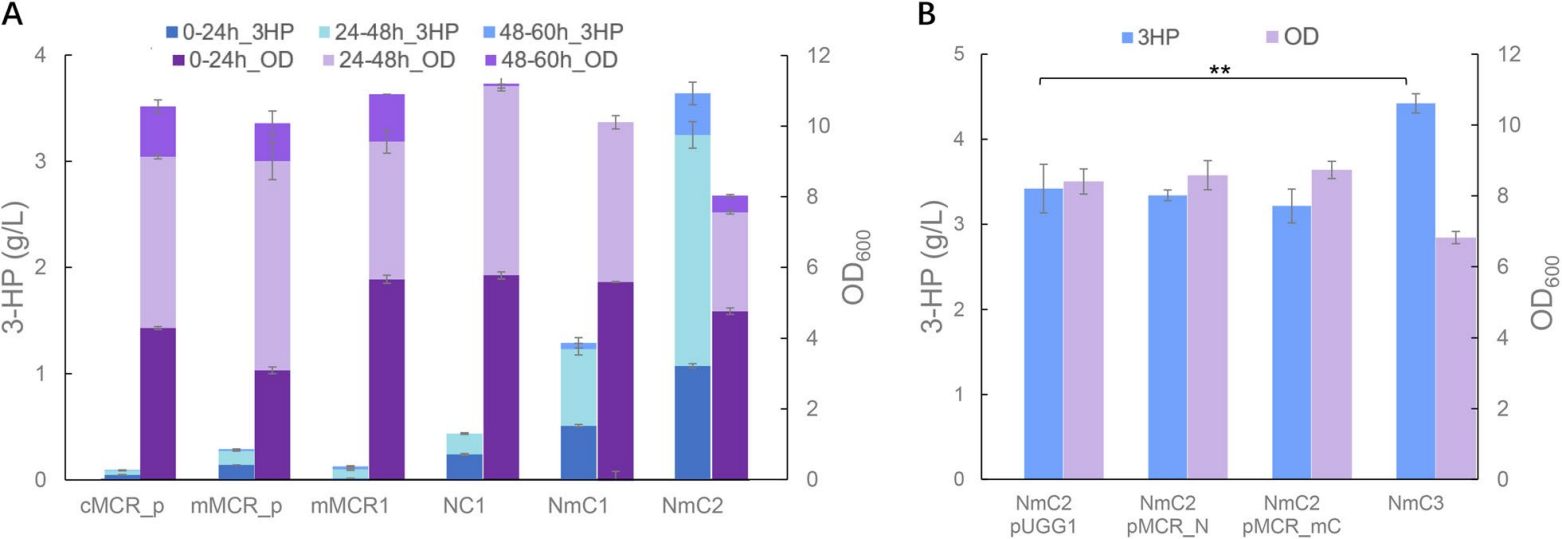
3-HP production in yeast mitochondria with the optimized expression of MCR genes. **A** Intact and dissected MCR gene expressed in the cytosol and mitochondria. cMCR_p, the strain harboring pYC01 with intact *MCR* targeted into the cytosol; mMCR_p, the strain harboring pMCR1 with intact *MCR* targeted into the mitochondria; mMCR1, the strain with intact *MCR* integrated at the site XI-3; NC1, the strain with *MCR-N* and *MCR-C* integrated at the site XI-3; NmC1, the strain with *MCR-N* and *MCR-mC* integrated at the site XI-3; NmC2, the strain with *MCR-N* and *MCR-mC* integrated at the sites XI-3 and X-4. **B** Varied expression of dissected *MCR-N* and *MCR-mC* in the mitochondria. NmC2 pUGG1, the strain NmC2 harboring an empty plasmid pUGG1; NmC2 pMCR_N, the strain NmC2 harboring pMCR-N; NmC2 pMCR_mC, the strain NmC2 harboring pMCR-mC; NmC3, the strain with *MCR-N* and *MCR-mC* integrated at the sites XI-3, X-4 and Int14. The cultivations were performed in biologically triplicate and error bars represent ± standard errors. Statistical analysis was performed using one-tailed Student’s *t* test (**p < 0.01)

In our previous research, we noticed that when the mitochondrial MCR was expressed in an episomal plasmid, the 3-HP titer reached 0.27 g/L [39], compared to the titer of 0.09 g/L when the cytosolic MCR was expressed [21], suggesting the yeast mitochondrion as an attractive subcellular compartment for 3-HP synthesis. With mitochondrial CaMCR integrated into the XI-3 site of the chromosome, the strain mMCR1 produced 0.11 g/L 3-HP, suggesting that CaMCR activity was crucial for 3-HP production. Therefore, dissected forms of CaMCR with higher enzymatic activities were then expressed in the mitochondria, including MCR-N&MCR-C, and MCR-N&MCR-mC with amino acid mutations. With the dissected genes integrated at XI-3 site, the constructed strains NC1 and NmC1 produced 3-HP at the titers of 0.43 g/L and 1.29 g/L, respectively (Fig. 2A). After a 2nd round integration of MCR-N & MCR-mC at the X-4 site in NmC1, the titer of the constructed strain NmC2 increased to 3.64 g/L, with its 70.6% accumulated during the ethanol phase, when the repressed mitochondrial activities were relieved. These results suggested that glucose derepression under glucose depletion conditions [49], contributed greatly to 3-HP synthesis in the presence of increased MCR activities. It also revealed that the MCR activity was a limiting factor for the mitochondrial 3-HP synthesis.

A previous study in *E. coli* showed that functional balance between MCR-N and MCR-mC could greatly enhance 3-HP synthesis by expressing three copies of MCR-N with chromosomal integration and MCR-mC in a high copy number plasmid. We then investigated whether altering the expression of MCR-N and MCR-mC could benefit 3-HP production in yeast mitochondria. However, neither of the constructed strains exhibited significant improvements in cell growth or 3-HP production (Fig. 2B), suggesting that the current expression of MCR-N and MCR-mC might be well-balanced (Additional file 3: Fig. S1). With a third integration of MCR-N and MCR-mC at the Int14 site of NmC2, the constructed strain NmC3 reached a titer of 4.42 g/L at 60 h, which was comparable with the highest titer achieved with the production in the yeast cytosol with a substantially engineered yeast chassis [18].

### Mitochondrial redox factor engineering for sufficient NADPH regeneration

Mitochondrial NADPH is generated from NADH by Pos5, whereas re-generation of NADPH from NADP^+^ is carried out by NADP^+^-dependent dehydrogenases including Ald4, Idp1 and Mae1 [33, 50, 51]. These enzymes were overexpressed under strong promoters to increase the availability of mitochondrial NADPH for 3-HP biosynthesis.

**Fig. 3:**
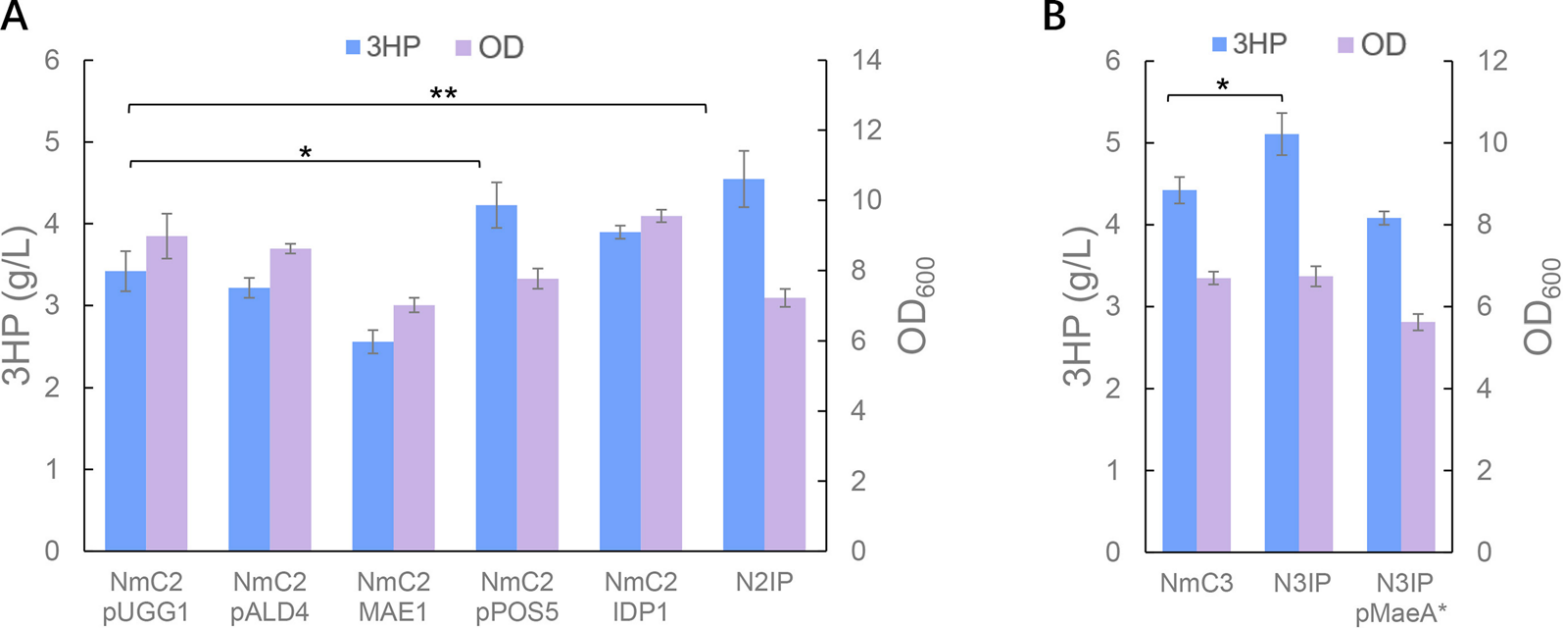
3-HP production with increased NADPH synthesis. **A** Overexpression of NADPH generating enzymes in the strain NmC2. NmC2 pALD4, the strain NmC2 harboring pALD4; NmC2 MAE1, the strain NmC2 with *MAE1* overexpressed; NmC2 pPOS5, the strain NmC2 harboring pPOS5; NmC2 IDP1, the strain NmC2 with *IDP1* overexpressed; N2IP, the strain NmC2 with *IDP1* and *POS5* overexpressed. **B** Overexpression of NADPH generating enzymes in the strain NmC3. N3IP, the strain NmC3 with *IDP1* and *POS5* overexpressed; N3IP pMaeA*, the strain N3IP harboring pMaeA*. The cultivations were performed in biologically triplicate and error bars represent ± standard errors. Statistical analysis was performed using one-tailed Student’s *t* test (*p < 0.05, **p < 0.01)

Indeed, when the plasmid pPOS5 for *POS5* overexpression was transformed into the strain NmC2, the 3-HP titer increased by 23.4%, while the biomass decreased by 13.6% (Fig. 3A). Yet, when *ALD4* was overexpressed in a high copy plasmid pALD4 into NmC2, no significant improvements were observed in 3-HP synthesis and cell growth. Similarly, when *MAE1* was overexpressed in NmC2 with its native promoter switched to a strong promoter *TDH3*p, 3-HP titer decreased from 3.42 to 2.56 g/L, which might be resulted from the decrease in cell growth. Nevertheless, with the native promoter replaced with a strong promoter *TEF1*p, *IDP1* overexpression in the strain NmC2 resulted in an increase in 3-HP production to 3.90 g/L, as well as a moderate improvement in cell growth. It has been reported that these dehydrogenases were transcriptionally regulated, and the varied effects of their overexpression on cell growth and 3-HP synthesis suggested that there might be other regulatory mechanisms involved in their activities.

With co-overexpression of *POS5* and *IDP1* in NmC2, the 3-HP titer of the constructed strain N2IP increased to 4.55 g/L, which was 33% higher than that of the strain NmC2. These results revealed that two routes for NADPH regeneration from NADH or NADP^+^ could synergistically boost 3-HP titer, with the one from NADH catalyzed by Pos5 as the main contributor. Then, both *IDP1* and *POS5* were overexpressed under strong promoters in the strain NmC3, generating the strain N3IP, with 3-HP titer increasing from 4.42 to 5.11 g/L (Fig. 3B).

However, overexpression of the native *MAE1* significantly slowed down cell growth and reduced 3-HP titer (Fig. 3A). To test whether the adverse result of *MAE1 *overexpression for 3-HP production is caused by its cofactor preference of NAD^+^ over NADP^+^, a mutated malic enzyme MaeA* that prefers NADP^+^ as the cofactor [52], was expressed in the mitochondria of the strain N3IP pMaeA*. Yet, the cell biomass and 3-HP titer still reduced by 17% and 20% (Fig. 3B), respectively. The negative effect on cell growth of malic enzyme overexpression might be that it drained TCA cycle intermediates and slowed down the operation of the TCA cycle, which provided essential energy for cell growth and maintenance.

### Limited flux of TCA cycle resulted in acetate overflow and impaired growth

**Fig. 4:**
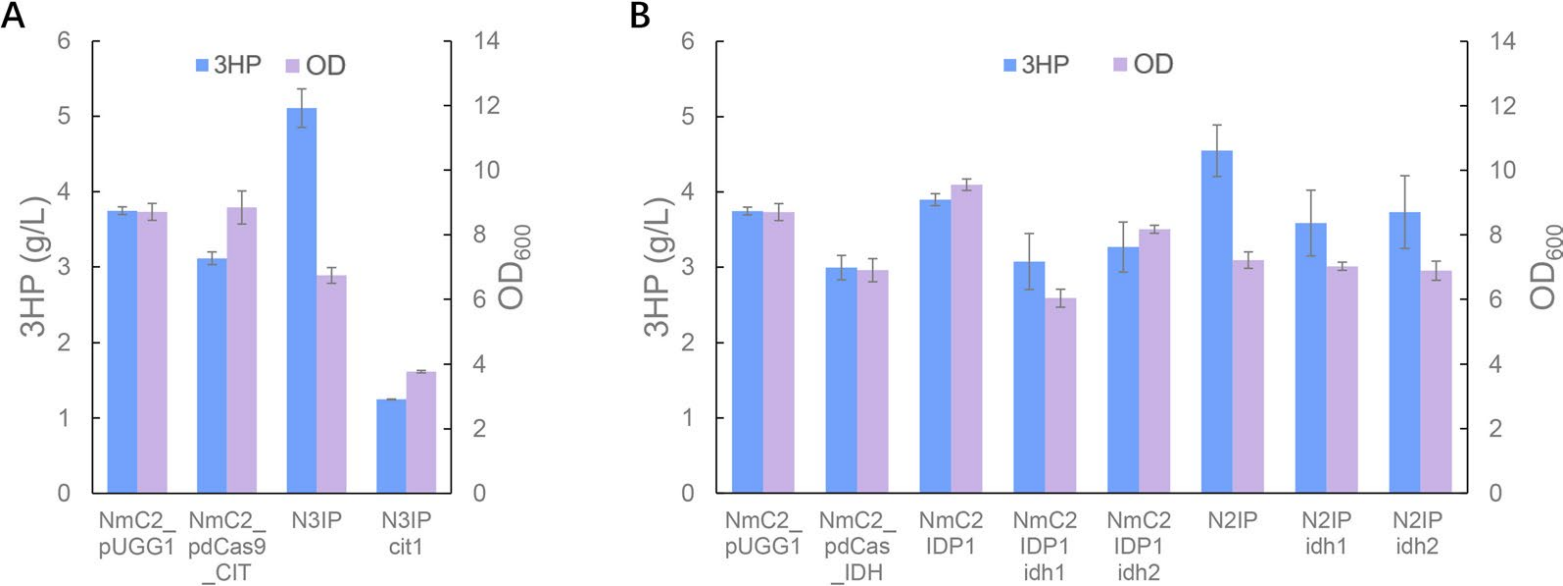
Limited flux of TCA cycle impaired cell growth and 3-HP production. **A** *CIT1* downregulation and deletion in strains NmC2 and N3IP. NmC2 pdCas9_CIT, the strain NmC2 harboring pdCas9_CIT; N3IP cit1, the strain N3IP with *cit1* deletion. **B** *IDH1/2* downregulation and deletion in NmC2 strains with and without *IDP1* and *POS5* overexpression. NmC2 pdCas_IDH, the strain NmC2 harboring pdCas_IDH; NmC2 IDP1 idh1, the strain NmC2 IDP1 with *idh1* deletion; NmC2 IDP1 idh2, the strain NmC2 IDP1 with *idh2* deletion; N2IP idh1, the strain N2IP with *idh1* deletion; N2IP idh2, the strain N2IP with *idh2* deletion. The cultivations were performed in biologically triplicate and error bars represent ± standard errors

Mitochondrial acetyl-CoA is dedicated to fuel the TCA cycle to provide energy for cell growth and maintenance. To redirect acetyl-CoA to 3-HP synthesis, attempts were made to limit the TCA cycle flux. The first reaction of the TCA cycle catalyzed by citrate synthase Cit1 is considered as the rate limiting step. Therefore, we first downregulated *CIT1* transcription by transforming a plasmid harboring dCas9 protein and two gRNAs targeting the *CIT1* promoter. However, *CIT1* downregulation resulted in a decrease in 3-HP titer from 3.75 to 3.11 g/L (Fig. 4A). When *CIT1* was deleted in the strain N3IP, the mutant showed significantly reduced growth and 3-HP production. The final biomass and 3-HP production were 56.0% and 24.4% of those in the strain N3IP, respectively. Meanwhile, acetate was accumulated during the cultivation of the mutant, which reached to 4.8 g/L (Additional file 3: Fig. S2). These results suggested that the disrupted TCA cycle resulted in acetate overflow, probably due to insufficient ATP production. The flux between 3-HP synthesis and the TCA cycle is, therefore, needed to be well-balanced.

Then another key enzyme of the TCA cycle, isocitrate dehydrogenase, that catalyzes the oxidation of isocitrate to alpha-ketoglutarate, was engineered to limit the TCA flux. Mitochondria contain both NAD^+^-dependent and NADPH^+^-dependent isozymes encoded by *IDH1/2* and *IDP1*, respectively. These two isozymes are regulated independently as they played different roles [53, 54], and both of them could tune the mitochondrial TCA activity [55, 56]. When the strain NmC2 was transformed with a plasmid harboring the dCas9 protein and gRNAs targeting *IDH1* and *IDH2* promoters, the mutant exhibited impaired cell growth with 3-HP production decreased by 20.7% and 20.1%, respectively (Fig. 4B). Similarly, in the strain NmC2 with *IDP1* overexpressed *IDH1* and *IDH2* deletion also decreased 3-HP production by 21.1%, and 16.2%, respectively (Fig. 4B). Meanwhile, a bigger biomass decrease observed in a *CIT1* deletion strain may have resulted from acetate overflow, which accumulated to 1.65 g/L in the culture. In the strain NmC2 with *IDP1* and *POS5* overexpressed, *IDH1* and *IDH2* deletion resulted in decreases in 3-HP titer by 21.1%, and 17.9%, respectively, yet no decreases in cell growth (Fig. 4B). Although *IDH1*/*2* downregulation and deletion resulted in impaired cell growth in the two strains, their deletion did not cause significant growth reduction in the strain with *IDP1* and *POS5* overexpression, probably because NADPH supply might still be the key factor for cell growth and 3-HP production in these strains.

### Fine-tuned acetyl-CoA carboxylase expression provided sufficient malonyl-CoA

**Fig. 5:**
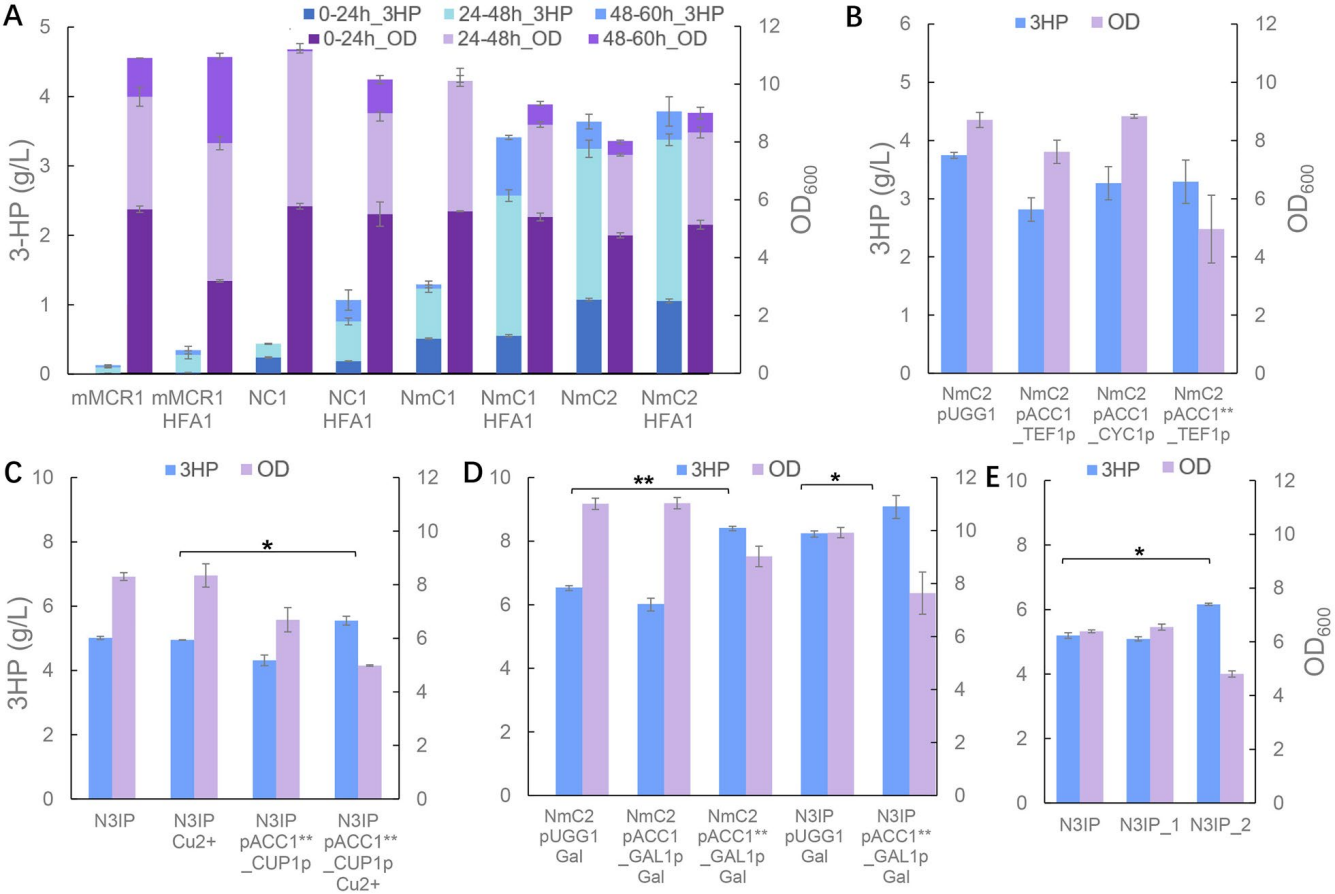
Overexpression of acetyl-CoA carboxylase in the mitochondria. **A** *HFA1* overexpression in the 3-HP producing strains. mMCR1 HFA1, the strain mMCR1 with *HFA1* overexpressed; NC1 HFA1, the strain NC1 with *HFA1* overexpressed; NmC1 HFA1, the strain NmC1 with *HFA1* overexpressed; NmC2 HFA1, the strain NmC2 with *HFA1* overexpressed. **B** *ACC1* and ACC1** overexpression in the stain NmC2. NmC2 pACC1_TEF1p, the strain NmC2 harboring pACC1_TEF1p; NmC2 pACC1_CYC1p, the strain NmC2 harboring pACC1_CYC1p; NmC2 pACC1**_TEF1p, the strain NmC2 harboring pACC1**_TEF1p. **C** Overexpression of ACC1** under the *CUP1* promoter with and without Cu^2+^ induction. N3IP Cu2 + , the strain N3IP with 300 μM Cu^2+^ supplemented at 24 h; N3IP pACC1**_CUP1p, the strain N3IP harboring pACC1**_CUP1p; N3IP pACC1**_CUP1p Cu2 + , the strain N3IP pACC1**_CUP1p with 300 μM Cu^2+^ supplemented at 24 h. **D** Overexpression of *ACC1* and ACC1** under the *GAL1* promoter with galactose induction. NmC2 pUGG1 Gal, the strain NmC2 pUGG1 with 20 g/L galactose supplemented at 24 h; NmC2 pACC1_GAL1p Gal, the strain NmC2 harboring pACC1_GAL1p with 20 g/L galactose supplemented at 24 h; NmC2 pACC1**_GAL1p Gal, the strain NmC2 harboring pACC1**_GAL1p with 20 g/L galactose supplemented at 24 h; N3IP pUGG1 Gal, the strain N3IP harboring pUGG1 with 20 g/L galactose supplemented at 24 h; N3IP pACC1**_GAL1p Gal, the strain N3IP harboring pACC1**_GAL1p with 20 g/L galactose supplemented at 24 h. **E** Overexpression of ACC1** in the strain N3IP_1. N3IP_1, the strain N3IP with *gal80* deletion; N3IP_2, the strain N3IP_1 harboring pACC1**_GAL1p_CEN. The cultivations were performed in biologically triplicate and error bars represent ± standard errors. Statistical analysis was performed using one-tailed Student’s *t* test (*p < 0.05, **p < 0.01)

Besides sufficient acetyl-CoA supply, sufficient malonyl-CoA supply is required for production of malonyl-CoA derived product, including cytosolic fatty acids and 3-HP expression [22, 27], as *ACC1* was stringently regulated in different levels. The regulation of the mitochondrial acetyl-CoA carboxylase Hfa1 has not been investigated in detail, and its impact on mitochondrial 3-HP production needs to be investigated. As shown in Fig. 5A, *HFA1* overexpression in the strains mMCR1, NC1 and NmC1 boosted 3-HP production by 2.65-fold, 1.63-fold, and 1.79-fold, respectively. These results revealed that *HFA1* overexpression greatly improved 3-HP synthesis during the ethanol growth phase (after 24 h). Yet, when *HFA1* was overexpressed in the strain NmC2, it could not significantly improve the 3-HP titer. The results indicated that *HFA1* might also be regulated to maintain the homeostasis of mitochondrial malonyl-CoA.

We, therefore, overexpressed *ACC1* in the mitochondria under the control of strong and weak promoters using *TEF1p* and *CYC1p*, respectively. Yet, impaired 3-HP synthesis was observed in both strains, with the overexpression under the strong promoter gave even lower titers compared with expression under the weak promoter (Fig. 5B), suggesting that *ACC1* overexpression might impose great burden, or it may also be regulated post-translationally in the mitochondria. Therefore, we overexpressed an Acc1 mutant ACC1** abolishing posttranslational regulations, yet the constructed strain exhibited similar production as the strain with *ACC1* overexpression but with impaired cell growth (Fig. 5B). Meanwhile, it was also observed that the strain with ACC1** overexpressed under *TEF1p* grew much slower, and the final 3-HP titer was achieved at 96 h compared with 60 h for other strains (Additional file 3: Fig. S3).

To release burden during early cell growth phase, ACC1** was then overexpressed in the mitochondria under the control of induced promoters *CUP1p* and *Gal1p*, respectively. In the strain N3IP, induced ACC1** expression with 300 μM Cu^2+^ supplemented at 24 h improved 3-HP titer from 5.01 to 5.50 g/L, while decreased 3-HP titer to 4.31 g/L without Cu^2+^ supplemented (Fig. 5C). The reduced biomass and 3-HP synthesis without Cu^2+^ supplemented at 24 h might be resulted from a basal expression as the medium contains 6 μM Cu^2+^ as a trace element. With the *GAL1* promoter and 20 g/L galactose supplemented at 24 h, ACC1** overexpression in the strains NmC2 and N3IP improved 3-HP titer by 28.7% and 10.3%, respectively, while *ACC1* overexpression failed to improve 3-HP synthesis (Fig. 5D). Then, *GAL80* was deleted in N3IP strain to release glucose regulation on the *GAL1* promoter [57]. In the constructed strain N3IP_2, the 3-HP titer increased by 18.6% to the final titer of 6.16 g/L (Fig. 5E), suggesting that fine-tuned ACC1* overexpression could coordinate malonyl-CoA requirements for cell growth and 3-HP synthesis, which thereof benefited 3-HP production in the mitochondria.

**Fig. 6:**
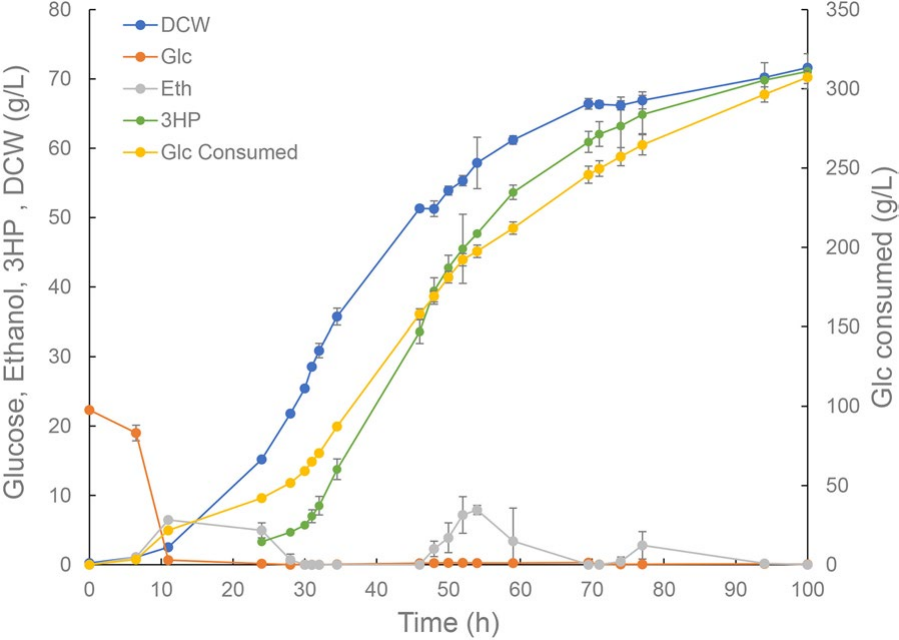
Fed-batch fermentation profiles of N3IP_2 under glucose limited conditions. Blue circles, dry cell weight (DCW); red circles, glucose concentration; grey circles, ethanol concentration; green circles, 3-HP titer; yellow circles, consumed glucose. The cultivations were performed in biologically duplicate and error bars represent ± standard errors

To further evaluate 3-HP production of the constructed strain, fed-batch cultivations were performed in 5 L bioreactors. To sustain the derepressed mitochondrial activity, continuous feeding was controlled under glucose limited conditions after 11 h, when glucose was almost exhausted (Fig. 6). The final 3-HP titer of 71.09 g/L was achieved at 100 h when the biomass reached 71.6 g/L, which was higher than the reported titer achieved through cytosol-based production so far. The 3-HP productivity reached 0.71 g/L/h, indicating its potential applications in industrial production. As shown in Additional file 3: Table S1, the titer and the productivity of N3IP_2 represented the highest values reported in *S. cerevisiae* via the malonyl-CoA pathway, suggesting that the yeast mitochondrion could be a suitable subcellular compartment for 3-HP production Yet, the yields on glucose of the strain N3IP_2 were 0.31 g/g and 0.23 g/g in the shake flask and fed-batch fermentation, respectively, compared with the maximum theoretical yield of 0.86 g/g. During the fed-batch cultivations, we observed that the 3-HP yield on glucose could reach as high as 0.40 g/g according to 3-HP production versus glucose consumption, suggesting that better feeding might improve 3-HP yields. Moreover, the yields could be further improved with better-tuned mitochondrial metabolism. As observed in this study, the flux distribution among the TCA cycle and 3-HP synthesis might require further investigation to balanced minimal growth requirements and maximum 3-HP production. In addition, it would be interesting to explore potential transporters involved in 3-HP transportation across the mitochondrial membranes and cytomembranes, as efficient transportation might relieve product inhibition and facilitate product synthesis.

## Conclusions

Metabolic modeling revealed that the malonyl-CoA pathway targeted into the mitochondria could synthesize 3-HP with a higher yield and a lower oxygen consumption. Overexpression of the mitochondrial MCR enzymes obtained the comparable 3-HP titer and yield with the highest level reported in the cytosol, indicating great potentials in harnessing the mitochondrial for 3-HP production. With further engineering on the mitochondrial NADPH metabolism and flux control at the acetyl-CoA node, the constructed strain obtained increased 3-HP titer by 39.4% in shake flask fermentations, and a final 3-HP titer of 71.06 g/L with a productivity of 0.71 g/L/h in fed-batch fermentations, the highest levels reported in *S. cerevisiae* so far. The high 3-HP productivity could be greatly credited for the controlled glucose limited conditions during the process, as the derepressed mitochondrial activities not only provide sufficient acetyl-CoA and cofactor but also finely tune flux toward malonyl-CoA for 3-HP synthesis.

## Supplementary Information

**Additional file 1: Table S1.** Plasmids, strains and primers constructed and used in the study.

**Additional file 2: Table S2.** Flux distribution from flux balance analysis with cytosolic MCR and mitochondrial MCR expressed.

**Additional file 3: Fig. S1.** Varied gene expression levels characterized by FPKM values. **Fig. S2.** Fermentation profiles of the strain N3IP cit1. **Fig. S3.** Fermentation profiles of the strain N3IP_pACC1**_TEF1p. **Table S1.** Summary of 3-HP bioproduction via malonyl-CoA pathway in *S. cerevisiae.*

## Data Availability

The data sets supporting the conclusions of the article are available within this article and its additional files. All data generated or analyzed during this study are included in this published article.

## Abbreviations

3-HP: 3-Hydroxypropionate
MCR: Malonyl-CoA reductase
ACC: Acetyl-CoA carboxylase
PDH: Pyruvate dehydrogenase complex
Hfa1: Mitochondrial acetyl-CoA carboxylase
Idp1: Mitochondrial NADP-specific isocitrate dehydrogenase
Pos5: Mitochondrial NADH kinase
Cit1: Mitochondrial citrate synthase
Aco1/2: Aconitase
Idh1/2: Mitochondrial NAD^+^ -dependent isocitrate dehydrogenase
Mdh1: Mitochondrial malate dehydrogenase
Mae1: Mitochondrial malic enzyme
EcMaeA*: Malic enzyme mutant from *E. coli*
