# Supplementary material for: Engineering yeast mitochondrial metabolism for 3-hydroxypropionate production
Source: Biotechnol Biofuels Bioprod. 2023 Apr 8;16:64. doi: 10.1186/s13068-023-02309-z (PMC10082987; doi:10.1186/s13068-023-02309-z)
Supplement: Supplementary file 3 — Additional file 3: Fig. S1. Varied gene expression levels characterized by FPKM values. Fig. S2. Fermentation profiles of the strain N3IP cit1. Fig. S3. Fermentation profiles of the strain N3IP_pACC1**_TEF1p. Table S1. Summary of 3-HP bioproduction via malonyl-CoA pathway in S. cerevisiae. [file 13068_2023_2309_MOESM3_ESM.docx]

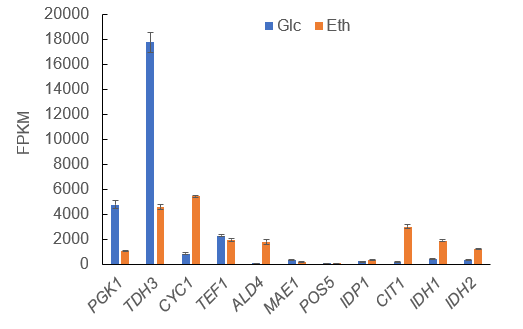


**Fig. S1** Varied gene expression levels characterized by FPKM values (Fragments Per Kilobase of exon model per Million mapped fragments) obtained from the transcriptional data from a previous study [1] when the wild type yeast cells were cultivated in glucose and ethanol medium in triplicate. The error bars represent ±standard errors.


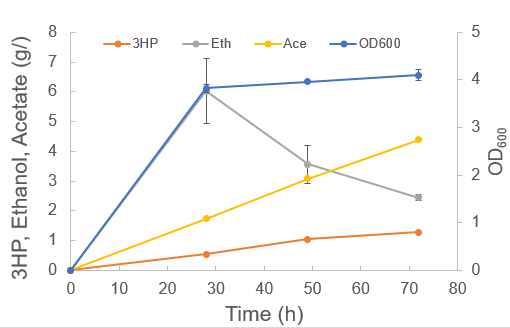


**Fig. S2** Fermentation profiles of the strain N3IP cit1. The cultivations were performed in biologically triplicate and error bars represent ±standard errors.


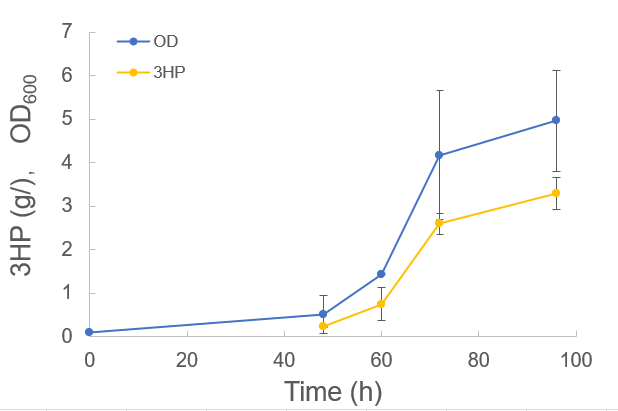


**Fig. S3** Fermentation profiles of the strain N3IP pACC1**_TEF1p (the strain N3IP harboring pACC**_TEF1p). The cultivations were performed in biologically triplicate and error bars represent ±standard errors.

**Table S1**. Summary of 3-HP bioproduction via malonyl-CoA pathway in *S. cerevisiae*

| Metabolic engineering strategies | Genetic modifications | Titers, yields, productivity | Reference |
| --- | --- | --- | --- |
| Improving malonyl-CoA supply | *MCR, ACC1^S6S9A S1157A^* | Bioreactor: 0.28 g/L, 0.014 g/gGlc | [2] |
| Improving acetyl-CoA, malonyl-CoA and NADPH supply | *MCR, HXT7p-ADH2, PGK1p-ALD6, mls1Δ, gapN, seACS^L641P^* | Shake flasks: 0.46 g/L, 0.023 g/gGlc | [3] |
| Improving malonyl-CoA supply with malonyl-CoA sensor | *MCR, PMP1* overexpression *or TPI1* overexpression | Shake flasks: ~1 g/L, ~0.05 g/gGlc | [4] |
| Dynamic modulation of pathway flux and enzyme expression levels | *TEF1p-BS123-MCR, HXT1p-FAS1, fapR* | Shake flasks (Feed beads): 1.0 g/L;  Fed-batch: 0.8 g/L, 0.054 g/gGlc | [5] |
| Improving acetyl-CoA, malonyl-CoA and NADPH supply | *MCR, ACC1^S659A,S1157A^, ALD6, seACS^L641^, PDC1, Tdh3::GAPDN* | Fed-batch: 9.8 g/L, 0.13 g/gGlc | [6] |
| Controlling the malonyl-CoA levels by manipulating the phospholipid synthesis transcriptional regulators | *MCR, ino2Δ* | Shake flasks: 0.48 g/L, 0.024 g/gGlc | [7] |
| Decoupling cell growth from product formation | *ICL1p-MCR* | Fed-batch: 0.58 g/L, 0.026 g/gGlc | [8] |
| Improving acetyl-CoA, malonyl-CoA and NADPH supply | *MCR, ACC1p::TEF1p, xPK-*  *PTA, ZWF1p::TDH3p, GND1p::PGK1p PGI1p::COX9, X-2::CCW12p-ALD6* | Shake flasks: 0.86 g/L, 0.043 g/gGlc | [9] |
| Optimizing the expression of MCR, Improving acetyl-CoA, malonyl-CoA and NADPH supply | *gal80∆, gal1∆, gal7∆, gal10∆, ’tesA∆, MmACL, RtME, MDH3, CTP1, TEF1p-ACC1; TEF1p-PYC1; MPC1, MPC3, AnACLa, AnACLb, RtCIT1, IDP2, YHM2, pgi1∆, PGI1, GND1, TKL1, TAL1, ZWF1, PGSY1-IDH2, XI-1::GAL7p-MCR-N-DIT1t, FAS1p::HXT1p, XII-3::TDH3p-MCR-N-FBA1t+DIT1t-MCR-C-TDH3p, XII-5::FBA1t-MCR-C-GAL1,10p-MCR-C-DIT1t* | Fed-batch: 56.5 g/L, 0.31 g/gGlc, 0.53 g/L/h | [10] |
| Mitochondrial targeting | CEN.PK 113-5D, *XI-3::pPGK1-CAT2m-(MCR-N)-tADH1_pTDH3-CAT2M-(MCR-C)-tCYC1* | Shake flasks: 0.43 g/L, 0.02 g/gGlc, 0.007 g/L/h | This study |
| Mitochondrial targeting, optimizing the expression of MCR | CEN.PK 113-5D, *XI-3::pPGK1-CAT2m-(MCR-N)-tADH1_pTDH3-CAT2M-(MCR-C***)-tCYC1* | Shake flasks: 1.29 g/L, 0.06 g/gGlc, 0.02 g/L/h | This study |
|  | NmC1, *X-4::pPGK1-CAT2m-(MCR-N)-tADH1_pTDH3-CAT2M-(MCR-C***)-tCYC1* | Shake flasks: 3.64 g/L, 0.18 g/gGlc, 0.06 g/L/h | This study |
|  | NmC2, *Int14::pPGK1-CAT2m-(MCR-N)-tADH1_pTDH3-CAT2M-(MCR-C***)-tCYC1* | Shake flasks: 4.42 g/L, 0.22 g/gGlc, 0.07 g/L/h | This study |
| Mitochondrial targeting, optimizing the expression of MCR genes, improving mitochondrial NADPH supply | NmC2, *pIDP1::pTEF1, pPOS5::pTDH3* | Shake flasks: 4.55 g/L, 0.23 g/gGlc, 0.08 g/L/h | This study |
|  | NmC3, *pIDP1::pTEF1, pPOS5::pTDH3* | Shake flasks: 5.11 g/L, 0.26 g/gGlc, 0.09 g/L/h | This study |
| Mitochondrial targeting, optimizing the expression of MCR genes, improving mitochondrial NADPH supply, regulating mitochondrial malonyl-CoA supply | N3IP, *gal80Δ*, pACC1**_GAL1p | Shake flasks: 6.16 g/L, 0.31 g/gGlc, 0.10 g/L/h;  Fed-batch: 71.09 g/L, 0.23 g/g Glc, 0.71 g/L/h. | This study |

**References:**

[1] Zhang Y, Su M, Wang Z, Nielsen J, and Liu Z. Rewiring regulation on respiro-fermentative metabolism relieved Crabtree effects in *Saccharomyces cerevisiae*. Synth Syst Biotechnol 2022; 7(4): 1034-1043.

[2] Shi S, Chen Y, Siewers V, and Nielsen J. Improving production of malonyl coenzyme A-derived metabolites by abolishing Snf1-dependent regulation of Acc1. MBio 2014; 5(3): e01130-14.

[3] Chen Y, Bao J, Kim IK, Siewers V, and Nielsen J. Coupled incremental precursor and co-factor supply improves 3-hydroxypropionic acid production in *Saccharomyces cerevisiae*. Metab Eng 2014; 22: 104-9.

[4] Li S, Si T, Wang M, and Zhao H. Development of a synthetic malonyl-CoA sensor in *Saccharomyces cerevisiae* for intracellular metabolite monitoring and genetic screening. ACS Synth Biol 2015; 4(12): 1308-15.

[5] David F, Nielsen J, and Siewers V. Flux Control at the Malonyl-CoA Node through Hierarchical Dynamic Pathway Regulation in *Saccharomyces cerevisiae*. ACS Synth Biol 2016; 5(3): 224-33.

[6] Kildegaard KR, Jensen NB, Schneider K, Czarnotta E, Ozdemir E, Klein T, et al. Engineering and systems-level analysis of *Saccharomyces cerevisiae* for production of 3-hydroxypropionic acid via malonyl-CoA reductase-dependent pathway. Microb Cell Fact 2016; 15: 53.

[7] Chen X, Yang X, Shen Y, Hou J, and Bao X. Increasing Malonyl-CoA Derived Product through Controlling the Transcription Regulators of Phospholipid Synthesis in *Saccharomyces cerevisiae*. ACS Synth Biol 2017; 6(5): 905-912.

[8] Maury J, Kannan S, Jensen NB, Oberg FK, Kildegaard KR, Forster J, et al. Glucose-Dependent Promoters for Dynamic Regulation of Metabolic Pathways. Front Bioeng Biotechnol 2018; 6: 63.

[9] Qin N, Li L, Ji X, Li X, Zhang Y, Larsson C, et al. Rewiring Central Carbon Metabolism Ensures Increased Provision of Acetyl-CoA and NADPH Required for 3-OH-Propionic Acid Production. ACS Synth Biol 2020; 9(12): 3236-3244.

[10] Yu W, Cao X, Gao J, and Zhou YJ. Overproduction of 3-hydroxypropionate in a super yeast chassis. Bioresour Technol 2022; 361: 127690.
